# Life-Threatening Respiratory Complications in Two Young Children with Extreme Obesity

**DOI:** 10.3390/children11121509

**Published:** 2024-12-11

**Authors:** Joanna Wielopolska, Klaudia Górnostaj, Joanna Olejnik-Wojciechowska, Maciej Kawczyński, Katarzyna Radomska, Elżbieta Petriczko

**Affiliations:** 1 Department of Pediatrics, Endocrinology, Diabetology, Metabolic Diseases and Cardiology, University Clinical Hospital No. 1, Pomeranian Medical University in Szczecin, 71-215 Szczecin, Poland; elzbieta.petriczko@pum.edu.pl; 2 Student Science Club KOLAR, Pomeranian Medical University in Szczecin, 71-215 Szczecin, Poland; 60968@student.pum.edu.pl; 3 Independent Social Nursing Laboratory, Pomeranian Medical University in Szczecin, 71-215 Szczecin, Poland; joanna.olejnik.wojciechowska@pum.edu.pl; 4 Department of Otolaryngology, University Clinical Hospital No. 1, Pomeranian Medical University in Szczecin, 71-215 Szczecin, Poland; maciej.kawczynski@pum.edu.pl (M.K.); katarzyna.radomska@pum.edu.pl (K.R.)

**Keywords:** obesity, children, consequences of obesity, obesity treatment, respiratory support

## Abstract

Background/Objectives: Obesity is a chronic disease characterized by pathological accumulation of adipose tissue. The exponentially increasing number of children with severe obesity draws attention to the tragic consequences of the lack of, or inadequate treatment of, obesity in this age group. This article aims to present ways of preventing obesity and ways of treating its complications in order to reduce the risk of the life-threatening problems caused by it. Case Report: The first patient was a 9-year-old boy with Prader–Willi syndrome, severe obesity, obstructive sleep apnea, hypertension, status post myocarditis, and recurring episodes of desaturation up to 70–80%. Respiratory support using continuous positive airway pressure (CPAP) and two-level positive airway pressure (BiPAP) were included in the treatment and the resolution of desaturation was observed. The second patient was a 5-year-old girl with simple obesity, obstructive sleep apnea, and subclinical hypothyroidism, hospitalized for sudden cardiac arrest, most likely caused by excessive fat tissue compressing the airway. Despite the introduced treatment, tracheostomy, and tonsillectomy, the girl remained unconscious during hospitalization and in the rehabilitation clinic, where she spent 7 months in a coma. Currently, her health is slowly improving as her weight significantly decreases. In both cases, serious consequences were observed due to non-adherence to dietary recommendations, lack of regular medical check-ups, and failure to implement appropriate treatment. Conclusions: Obesity can lead to life-threatening consequences, including respiratory arrest and a need for respiratory support, if proper treatment is not administered and if medical recommendations are not followed.

## 1. Introduction

Obesity is a chronic, recurrent disease associated with the pathological accumulation of adipose tissue [1].

According to WHO, up to the age of 5 years, obesity is diagnosed when the ratio of body weight to body height is >3 standard deviations above the median [2]. Polish centile grids for BMI are used in children aged 3–18 years, where obesity is diagnosed >2 standard deviations (>97 percentile) [3]; alternatively, in children aged 5–19 years, WHO standards can be used with the same definition [2]. In addition, due to the increasing risk of observed complications with increasing weight, the diagnosis of severe or class III obesity defined as a BMI exceeding 3 standard deviations from the median (>99.9 percentile) is used [2]. The prevalence of obesity in children is increasing dramatically. Between 1975 and 2016, there was an eightfold increase in the incidence of the disease in the age group of 5 to 19 years [4].

The etiology of simple obesity is a positive energy balance, i.e., an excessive amount of energy supplied compared to the amount of energy expended [5]. The primary factors leading to its development are poor eating habits, insufficient physical activity, a sedentary lifestyle, sleep restriction, and abnormal eating behaviors, such as overeating as a way of coping with emotional tension. Obesity can also be secondary. In this case, it is not connected only with increased estimated energy requirement, but associated with endocrine disorders (hypothyroidism, hypercortisolemia, and growth hormone deficiency), medications taken (corticosteroids, valproic acid, and atypical neuroleptics, most notably, risperidone) and genetic disorders such as Prader–Willi syndrome [6].

Prader–Willi syndrome is the most common cause of syndromic obesity, occurring with a frequency of 1:15,000–1:25,000 births. In most cases (70%), it is associated with a deletion of the 15q11–13 region of the paternally derived chromosome. A Holm scale created in 1991 is useful for clinical diagnosis, and cytogenetic testing is necessary for confirmation. The characteristic features of the syndrome are as follows: muscular hypotonia in the neonatal period resulting in difficulty in latching and small body gains, excessive appetite, lack of satiety, and rapid weight gain after 2 years of age leading to central obesity, features of facial dysmorphia such as narrow face, almond-shaped eyelid crevices, small mouth, thin upper lip, corners of the mouth pointing downward, small hands and feet, and hormonal deficiencies resulting in hypogonadism, growth hormone deficiency, and hypothyroidism. Some patients present with an intellectual disability [7].

The primary objective of obesity treatment in younger children, defined as those up to the growth spurt period occurring during puberty, is to achieve weight stabilization with BMI reduction accompanying height gain. In older children, defined as those from the final pubertal period onward, the objective is to reduce weight by 1–2 kg per month [8]. Therapy is based on strict adherence to dietary recommendations associated with a permanent change in diet, taking into account the principles of healthy eating, and regular physical activity using mainly aerobic exercise. Psychological support is useful in therapy. The introduction of pharmacological treatment should be considered when the expected results have not been achieved despite the use of the methods listed above [9]. The only drug registered in Poland for the treatment of obesity in children is liraglutide, an analog of human glucagon-like peptide 1, which can be used in children aged 12 years and older with a BMI equivalent to ≥30 kg/m^2^ in adults or with a body weight > 60 kg [10]. Bariatric treatment can also be used in selected cases [11].

The treatment of obesity in Prader–Willi syndrome is based on similar principles. It is important to introduce principles of healthy nutrition from infancy before hyperphagia appears. At the first signs of uncontrollable appetite, it is essential to limit the size and caloric content of the meals consumed. Muscular hypotonia obliges early initiation of rehabilitation. A distinction in Prader–Willi-related obesity treatment is the possibility of therapy with recombinant human growth hormone, which results in improved metabolic parameters, reduced body fat mass, increased muscle strength, and improved respiratory capacity. It is subjected to strict indications for its inclusion, such as nutritional status as measured by a BMI below the 97th percentile for age and sex, and compensated carbohydrate disorders [7]. Bariatric surgery is contraindicated in patients with Prader–Willi syndrome due to intellectual impairment, which precludes the ability to give informed consent to the procedure and may cause difficulties in adhering to postoperative recommendations [6].

The development of complications is observed if obesity is not addressed early enough. The most common are hypertension, prediabetes, diabetes type 2, and dyslipidemia [6].

One of the groups of complications is respiratory dysfunction. They are characterized primarily by the occurrence of obstructive sleep apnea syndrome (13–59% of overweight or obese children) associated with shallowing or lack of airflow through the upper airway with normal chest movements leading to reduced airflow and hypoxia [6]. The occurrence of obstructive sleep apnea syndrome is a risk factor for cardiovascular complications and hypertension and a cause of behavioral disorders, learning difficulties, memory impairment, and deterioration of quality of life, among others [12]. A polysomnographic study is used for diagnosis. Treatment includes weight reduction, and in severe obstructive sleep apnea syndrome, therapy is based on the use of non-invasive ventilation (NIV) and continuous positive airway pressure (CPAP). In addition, people with obesity are more likely to have asthma, compared to normal-weight patients, worse respiratory parameters are found on spirometry, and the treatment is less effective [6].

Other complications of obesity can be gastrointestinal disorders (represented mainly by metabolic dysfunction-associated steatohepatitis, and, in addition, gallstones and gastroesophageal reflux are also present), polycystic ovary syndrome, precocious puberty, hyperandrogenism in girls, osteoarticular disorders (juvenile desquamation of the femoral head, Blount’s disease, valgus knees, spinal pain, increased risk of fractures, and flat feet), renal dysfunction, neurological disorders (migraine and idiopathic intracranial hypertension) and mental health disorders [6]. Failure to undertake proper treatment of obesity and the development of its complications leads to a risk to the child’s health and life. Additional increases in blood pressure and depressive and breathing disorders are life-threatening complications, which are emergency situations.

## 2. Clinical Cases

### 2.1. First Clinical Case

A 9-year-old patient with Prader–Willi syndrome was admitted to the Department of Pediatrics, Endocrinology, Diabetology, Metabolic Diseases and Cardiology of the University Clinical Hospital No. 1 of the Pomeranian Medical University in Szczecin for further diagnosis and treatment of complications of the disease. The boy came from a third pregnancy, third birth, and was born by cesarean section at 38 weeks of gestation with a birth weight of 2230 g, rated 7/8/8 points on the Apgar scale. After birth, he was diagnosed with decreased muscle tone, ineffective suckling, and scrotal hypoplasia. In 2014, when the boy was two weeks old, he was hospitalized at the Clinic for perinatally identified problems. During the consultation, numerous dysmorphic features were described—narrow upper lip, corners of the mouth turned downward, almond-shaped eyes, gothic palate, scrotal hypoplasia, absence of testes in the scrotum, pale skin, and light hair color. The entire clinical picture, together with the laboratory tests performed, pointed to a diagnosis of Prader–Willi syndrome, which was confirmed by genetic testing.

The boy remained out of the Clinic’s care for nine years due to a difficult family situation. Despite the efforts of doctors, dietitians, and nurses, the mother did not follow dietary recommendations and did not attend recommended medical checkups with the child.

At the age of 9, the child was burdened with severe obesity (weight 73.5 kg (>97 percentile), height 137 cm (50 percentile), and BMI 39.16 kg/m^2^ (>99.9 percentile)), as well as obstructive sleep apnea. At that time, the boy was hospitalized in the Infectious Diseases Unit for suspected hepatitis (elevated aminotransferases), weakness, and bronchitis caused by Mycoplasma pneumoniae. Subsequently, due to his deteriorating general condition and elevated troponins and NT-proBNP levels, the boy was transferred to the Pediatric Cardiology Unit, where myocarditis was diagnosed. In addition, the boy developed hypertension. Treatment included furosemide, amlodipine, ramipril, fusidic acid, cefazolin, cefuroxime, azithromycin, nystatin, furagin, salbutamol and albumin. During hospitalization for obstructive sleep apnea, there were numerous episodes of apnea and desaturation of down to 70–80%. In the hospital ward, the child slept in a semi-sitting position and required passive oxygen therapy at night.

After his clinical condition improved, the boy was transferred to the Department of Pediatric Endocrinology for further treatment of metabolic disorders and sequelae of Prader–Willi syndrome. Laboratory tests performed on admission showed leukocytosis with granulocytosis, elevated C-reactive protein (CRP) levels, a downward trend in aminotransferase levels, and normalization of cardiac parameters. Given the observed increase in CRP, empirical antibiotic therapy with amoxicillin–clavulanic acid was administered. Several microbiological tests were performed, which revealed Staphylococcus hominis MRCNS cultured from central venous access, so antibiotic therapy was modified to include vancomycin.

Due to repeated episodes of apnea, especially during sleep, with saturation below 80%, passive oxygen therapy was initially used. The boy was consulted repeatedly by anesthesiologists, as well as otorhinolaryngologist (ENT) specialists. An ENT examination revealed enlargement of the palatine tonsils in Brodsky grade II, meaning tonsils exceeding the lines of the palatal arches, occupying between 26 and 50% of the width of the pharynx.

A high-flow intranasal oxygen therapy with non-invasive ventilation (HFNc+NIV) assisted breathing system was applied using the OptiFlow kit, with mediocre tolerance from the patient. Subsequently, the treatment was changed to BiPAP breathing support with good clinical results.

The patient was initially qualified for tonsillectomy due to myocarditis, but it was postponed due to persistently elevated inflammatory markers. He was also consulted by a cardiologist—neither echocardiography nor 24 h blood pressure monitoring showed contraindications to general anesthesia. During a subsequent ENT consultation, the patient was scheduled for an elective tonsillectomy once the inflammation had resolved.

Subsequent studies showed a downward trend in CRP levels. It was hypothesized that the constantly elevated CRP index persists in the patient due to chronic hypoxia in the course of apnea and chronic inflammation caused by class III obesity. During hospitalization at the Clinic, a low-calorie diet was implemented, which reduced the boy’s weight by 5 kg.

The boy was discharged home in good general condition with the following recommendations: continuation of amlodipine, furosemide, and ramipril; the need to follow dietary recommendations and continue to strive for weight reduction; rehabilitation; the prevention of sleep-disordered breathing using the BiPAP device; and further ENT, endocrine, metabolic and cardiac care.

During the patient’s follow-up after leaving the Clinic, steady weight reduction and clinical improvement were observed, proving the necessity of adhering to recommendations and striving for normal weight to reduce the risk of life-threatening complications.

### 2.2. Second Clinical Case

The second case involves a 5-year-old girl burdened with severe obesity diagnosed in infancy, sleep apnea syndrome, and subclinical hypothyroidism. The patient had previously been hospitalized in the Infant Department of the Department of Pediatrics, Endocrinology, Diabetology, Metabolic Diseases, and Cardiology of the Developmental Age. Information collected from parents revealed numerous dietary errors, but after discharge, the parents did not follow the recommendations and regular medical checks.

**Figure 1:**
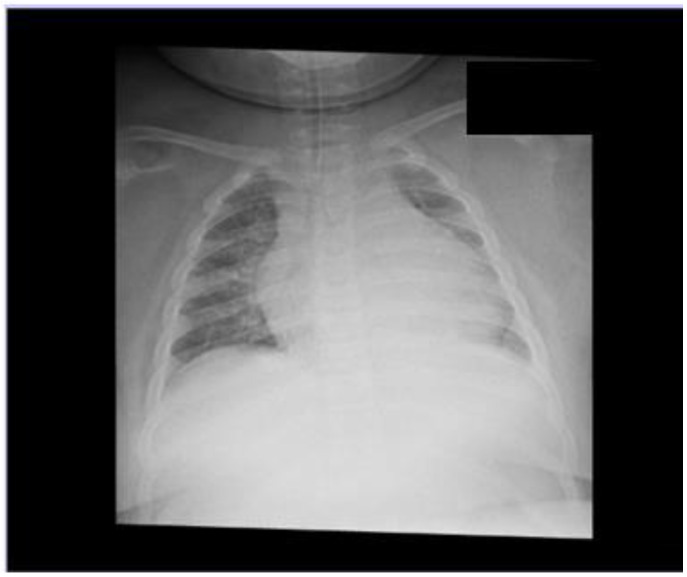
Bedside chest x-ray in supine position. Left lung obscured by the shadow of a greatly enlarged heart.

The child suffered sudden respiratory arrest and subsequent cardiac arrest while at home in the presence of her parents. The girl’s mother realized that the child was not breathing after a night’s sleep, so it is difficult to assess how long the CNS hypoxia lasted. After a successful two-minute resuscitation by the Emergency Medical Services Team, the patient was admitted to the Intensive Care Unit in critical condition, graded 3 on the Glasgow scale. Saturation at the time fluctuated between 70% and 100%. Initially, a BiPAP machine was used for respiratory support, but due to sudden desaturation to 20% with bradycardia to 50 beats/min, atropine, and dexamethasone were included in the treatment, and ventilation with AMBU was applied, with improvement in saturation. After 6 h, deep desaturation occurred again, a bolus of cisatracurium and sedative drugs was administered without improvement in airway pressures, an advanced mode of mechanical ventilation (APRV) was attempted, a maximum saturation of 94% was achieved, and hypercapnia persisted. The girl was sedated and treated with neuroprotective lidocaine and antiedema with 3% NaCl. A head CT scan, ECG, and cardiac ultrasound showed no abnormalities. ENT consultation revealed a Brodsky grade II hypertrophy of the palatine tonsils. On physical examination, attention was drawn to significant abdominal obesity (body height: 120 cm (97 percentile), weight: 87 kg (>97 percentile), BMI: 60.4 kg/m^2^ (>99.9 percentile)) making examination difficult, and prominent cafe au lait spots on the skin of the extremities. In the first 24 h of hospitalization, the child’s condition deteriorated, the patient was unconscious under quadrivalent sedation (Fentanyl + Midanium + Propofol + Ketamine), the pupils were equal and reactive, and truncal reflexes were preserved. Chest X-ray (Figure 1) showed atelectasis-inflammatory changes in the right lung. The left lung was obscured by the shadow of a significantly enlarged heart. Lung ultrasound was impossible to evaluate due to abundant adipose tissue, and small subpleural consolidations and single B-lines were visible in the accessible portions of the lung in the axillary lines. Auscultation revealed symmetrical rales and wheezes. Mechanical ventilation was significantly impaired, but with ventilation with 100% oxygen and saturation of up to 89%, it was possible.

On the following day, there were drops in saturation to 60% during the suctioning of airway secretions and spontaneous drops to 85%. Continuous infusion of rocuronium did not improve mechanical ventilation conditions. The patient was subsequently ventilated in very different modes, with a reversed inspiration/expiration (I:E) ratio, and recruitment maneuvers were performed—with no improvement. There was an episode of dyssynchrony with the ventilator despite four-mode sedation and rocuronium infusion, and a drop in SpO_2_ to 60% with a very slow return of SpO_2_ to 80% after therapeutic intervention.

The patient underwent a tracheotomy procedure due to the expected prolonged and difficult ventilation. Obtaining access to the trachea was difficult due to excess subcutaneous fatty tissue, but anesthesia and the procedure went without complications. A 6.0 tracheostomy tube was placed with a cuff to the full depth of the tube. After returning from the operating theater, the patient was connected to a ventilator in Duo PAP mode with FiO_2_ 0.95, and placed in a semi-sitting position. Sedation was reduced, and the rocuronium infusion was discontinued. After about two hours, the patient was observed to turn on her own respiratory drive, with a deepening of tidal volume, followed by the intensification of the cough reflex and desaturation to 45%.

Despite the deep position of the tracheostomy tube and concern about its protrusion, the decision was made to perform a life-saving maneuver—inversion to the prone position. Sedation was again deepened, and the patient was transferred to a gutter to decompress the monstrous abdomen. A dorsal lung ultrasound was performed—evaluation was very difficult due to the 5 cm layer of fat—significant atelectasis and subpleural consolidations were visualized on the left side and only subpleural changes were on the right.

After the transfer to a prone position and return to sedation (Midanium, Ketamine, Fentanyl, Propofol, and rocuronium), there was a significant improvement in saturation to 97%. All attempts to reduce inspiratory pressure, positive end-expiratory pressure, and the number of breaths resulted in a significant decrease in the minute volume, a drop in saturation, and a rise in etCO_2_ to as high as 90 mmHg.

Deterioration of ventilation was also observed in the lateral position, so the patient was returned to the prone position. BiPAP mechanical ventilation was maintained in this position. Auscultation revealed a clear alveolar murmur without additional auscultatory phenomena, saturation was maintained at 98%, EtCO_2_ 45, and the patient was gasometrically equilibrated.

Mechanical ventilation in the supine position with the headrest elevated to 30 degrees was ineffective in previous settings. The prone position was used twice a day for 6–8 h.

On the third day of hospitalization, the patient was described as in respiratory failure and hemodynamically unstable. Sustaining respiratory function with ventilator therapy was difficult to achieve due to inflammation and congestion in the lungs and a large fat mass weighing down the chest and impairing its mechanics.

During the following days, the saturation was maintained at 98% in the semi-sitting position; it dropped to 91% during the flat position for the head MRI. Ventilation by tracheostomy using a ventilator was continued throughout hospitalization.

During hospitalization, the patient underwent an adenotomy (the pharyngeal tonsil was enlarged to grade I/II, i.e., partially obscuring the posterior nostrils, no more than 50%) to dilate the burdened airway. However, it cannot be concluded that the SCA and the girl’s severe condition were caused by tonsil hypertrophy.

It was determined that the respiratory arrest occurred due to compression of the structures of the respiratory system caused by class III obesity. Respiratory distress caused by extreme obesity can lead to serious consequences, including sudden cardiac arrest, which is immediately life-threatening. During extreme obesity, the airways may be compressed by excess adipose tissue, which may imply the need to remove the undersized tonsils to dilate the burdened upper airways. This condition is particularly dangerous in children due to the immaturity of the musculoskeletal system, which may not be able to compensate for such a large mass pushing down on the chest and neck. This case report proves the importance of maintaining adequate weight in children.

The patient remained unconscious until the end of her hospital stay and later when she was transferred to the Budzik Clinic in Warsaw. She had been in a coma for 7 months when she slowly started to regain consciousness. According to the patient’s mother, at first, she was reacting to voice, then she began to move voluntarily, and, currently, she is an active girl, who can walk, play with puzzles, and understand what is said to her. She is unable to talk yet as she still has a tracheostomy, although she does not need mechanical respiratory support. During her stay in the Budzik Clinic, the patient was under the care of a clinical dietitian and lost 54 kg (current measurements at 6 years and eight months of age—body height: 130 cm (90–97c), weight: 33 kg (97c), BMI: 19.5 kg/m^2^ (97c)). The patient is at home, and the tracheostomy was closed. She is currently treated with baclofen, amantadine, levetiracetam, quetiapine, omeprazole, and weekly injections of cerebrolysin. The extent of the neurocognitive damage is yet unknown. It is worth noticing that even though the patient lost 54 kg, she is still considered obese and requires further dietary restrictions, discipline, and medication in order to recover after the cardiac arrest incident and stay in good health.

## 3. Discussion

According to the data published in 2021, 700,000 Poles suffer from class III obesity, and it is a problem that is often underestimated [13]. In Poland, a social campaign called “In the New Shape” has been established to spread awareness and help fight against severe obesity [14]. Obesity has a significant impact on increasing the incidence of distant complications in the form of dyslipidemia, acute exacerbations of obstructive sleep apnea, diabetes, hypertension, non-alcoholic fatty liver disease, or weight-related arthropathy [15]. The presence of multiple comorbidities with obesity during development significantly reduces children’s quality of life and increases the risk of depression [16]. This disease found in children is difficult to treat, and treatment programs often have short-term effects. Therefore, prevention plays an extremely important role in terms of reducing the prevalence of obesity. Promoting a healthy lifestyle, sports and a proper diet are irreplaceable elements that should involve not only school institutions, but especially the family. When non-invasive techniques fail, it is worth considering bariatric surgery, which offers a long-term effect and the chance to implement lifestyle-improving measures for young patients at that time [17].

In the case of the girl described, the parents insisted that their daughter’s obstructive sleep apnea was caused by hypertrophied tonsils. Prior to the episode of sudden cardiac arrest, the girl had been consulted with an ENT doctor for tonsillar hypertrophy, but an examination during hospitalization showed tonsillar hypertrophy of Brodsky grade II (tonsils slightly exceeding the palatal arches), and therefore it could not be the sole cause of sudden cardiac arrest. The parents found it very difficult to accept that the cause of the girl’s critical condition was obesity.

The available literature has highlighted the importance of parental education on non-pharmacological treatment of childhood obesity. The results of a study are described, in which a statistically significant decrease in insulin resistance was achieved in children whose mothers attended six educational meetings on the nutrition of obese children relative to children whose mothers did not receive such training (but received standard medical advice) [18]. In Szczecin, there is an educational program called “Prevention of overweight and obesity among children aged 8 attending elementary school in Szczecin—Brave Eight”. It aims to improve the health of second-grade students of elementary schools in the Szczecin area by reducing the risk of overweight and obesity and other civilizational diseases. The program includes medical, psychological, dietary, and physical activity specialist consultations, as well as workshops for parents, children, teachers, and school nutrition staff. The need for extensive preventive measures has been observed, given the excessive body weight diagnosed in 24.1% of the population surveyed [19]. Furthermore, the German KIGGS study described in 2018 was created in reference to the first phase conducted in 2007. It indicated that the overweight trend is stable but still high, which emphasizes the need for further preventive actions. Overweight was diagnosed in 15.4% and obesity in 5.9% of children [20]. In addition, several disorders of the relationship with food in children that can lead to severe obesity have been described. In the available article, they are highlighted as follows: emotional eating (EE); binge eating disorder (BED)—most common in boys; Bulimia Nervosa (BN)—most common in girls; night eating syndrome (NES); and sleep-related eating disorder (SRED) [21]. In the case of the boy described, the problem most likely relates to a lack of satiety sensation, typically described in Prader–Willi syndrome, causing him to constantly demand food, but the patient had no support from his family environment. Another type of disorder was the basis of obesity in the girl, who at her peak weighed 87 kg at a height of 120 cm, so her relationship with food was certainly not normal and probably included at least one of the mentioned disorders. In the articles describing the use of respiratory support with the BiPAP system, the authors emphasize that tracheotomy in children should be considered a treatment of last resort. Maintaining a physiological airway in pediatric patients is particularly important, due to the development of the body and speech [22]. BiPAP maintains positive airway pressure during both inspiration and expiration, allowing airways burdened by excessive fatty tissue to remain expanded during sleep. This system is also used in other conditions such as bronchial asthma, and is also a very valuable tool in protecting the airway in obstructive sleep apnea, which was present in the patients described [23]. In the boy, fat tension on the airway began to cause obstructive sleep apnea, but therapy with BiPAP proved effective, and saturation at night remained normal. After several days of using the BiPAP system during the night, the child’s daytime activity improved significantly. In addition to the other benefits of this treatment (improved mood, better focus while studying), the boy can exercise, which, combined with adherence to the recommended diet, is the basis of the non-pharmacological treatment of obesity. It should be emphasized that persistently elevated inflammatory parameters were a contraindication to general anesthesia. After excluding a potential source of infection, it was found that it could be related to the boy’s severe obesity.

Unfortunately, in the case of the described girl, the BiPAP mask was not enough to maintain saturation at an appropriate level. Most likely, the excessive mass of hypertrophied adipose tissue limited the ability to maintain positive upper airway pressure even with the BiPAP system. Due to continuous drops in saturation down to 60% and the prospect of the need for prolonged mechanical ventilation (the patient had remained unconscious since the onset of sudden cardiac arrest), we decided to perform a tracheotomy. In the girl’s case, a genetic syndrome was excluded.

## 4. Conclusions

The cases of two children with severe obesity described in the article, despite their different causes, show how important it is to prevent the development of excessive body weight. Complications that develop in children with obesity can significantly affect the quality and length of their lives, but it is difficult to find information about cases of death in children directly caused by simple severe obesity [24], although, in the case of the described girl, it contributed to a sudden cardiac arrest [25].

In some children with Prader–Willi syndrome, recombinant growth hormone therapy may improve the prognosis of severe obesity [26]. Actions are necessary to early identify children at risk of respiratory disorders due to obesity.

## Data Availability

No datasets were generated or analyzed during the current study.
